# Supercritical Fluid Extraction of Fucoxanthin from the Diatom *Phaeodactylum tricornutum* and Biogas Production through Anaerobic Digestion

**DOI:** 10.3390/md20020127

**Published:** 2022-02-07

**Authors:** Mari Carmen Ruiz-Domínguez, Francisca Salinas, Elena Medina, Bárbara Rincón, Marí Ángeles Martín, Marí Carmen Gutiérrez, Pedro Cerezal-Mezquita

**Affiliations:** 1 Laboratorio de Microencapsulación de Compuestos Bioactivos (LAMICBA), Departamento de Ciencias de los Alimentos y Nutrición, Facultad de Ciencias de la Salud, Universidad de Antofagasta, Antofagasta 1240000, Chile; francisca.salinas@uantof.cl (F.S.); elena.medina.perez@ua.cl (E.M.); 2 Instituto de la Grasa, Consejo Superior de Investigaciones Científicas (CSIC), Campus Universidad Pablo de Olavide, Edificio 46. Ctra. de Utrera km. 1, 41013 Seville, Spain; brlloren@cica.es; 3 Departamento de Química Inorgánica e Ingeniería Química, Universidad de Córdoba, Campus Universitario de Rabanales, 14071 Córdoba, Spain; iq2masam@uco.es (M.Á.M.); a12gumam@uco.es (M.C.G.); 4 Instituto de Química Fina y Nanotecnología (IUNAN), Universidad de Córdoba, Campus de Excelencia Internacional Agroalimentario CeiA3, Edificio Marie Curie (C-3), Ctra. N-IV, km 396, 14071 Córdoba, Spain

**Keywords:** *Phaeodactylum tricornutum*, supercritical fluid extraction, co-solvent, fucoxanthin, anaerobic digestion, algae biorefinery

## Abstract

*Phaeodactylum tricornutum* is the marine diatom best known for high-value compounds that are useful in aquaculture and food area. In this study, fucoxanthin was first extracted from the diatom using supercritical fluid extraction (SFE) and then using the extracted diatom-like substrate to produce bioenergy through anaerobic digestion (AD) processes. Factors such as temperature (30 °C and 50 °C), pressure (20, 30, and 40 MPa), and ethanol (co-solvent concentration from 10% to 50% *v/v*) were optimized for improving the yield, purity, and recovery of fucoxanthin extracted using SFE. The highest yield (24.41% *w/w*) was obtained at 30 MPa, 30 °C, and 30% ethanol but the highest fucoxanthin purity and recovery (85.03mg/g extract and 66.60% *w/w*, respectively) were obtained at 30 MPa, 30 °C, and 40%ethanol. Furthermore, ethanol as a factor had the most significant effect on the overall process of SFE. Subsequently, *P.tricornutum* biomass and SFE-extracted diatom were used as substrates for biogas production through AD. The effect of fucoxanthin was studied on the yield of AD, which resulted in 77.15 ± 3.85 LSTP CH_4_/kg volatile solids (VS) and 56.66 ± 1.90 LSTP CH_4_/kg VS for the whole diatom and the extracted *P.tricornutum*, respectively. Therefore, *P.tricornutuman* can be considered a potential source of fucoxanthin and methane and both productions will contribute to the sustainability of the algae-biorefinery processes.

## 1. Introduction

Diatoms are unicellular photosynthetic eukaryotes that are commonly found in marine ecosystems and moist terrestrial habitats. They contribute to about 20–25% of global carbon dioxide fixation through photosynthesis and play an important role in the global silicon cycle [1,2]. In addition, they are a part of the base of marine trophic networks worldwide and contribute to at least 20% of annual primary productivity, which is equivalent to tropical forests [3,4]. Among the organic molecules produced by diatoms, fatty acids or carotenoids are essential in the nutrition of benthic and pelagic animals [5]. Recently, novel and green applications have been discovered from intracellular molecules present in diatoms such as total lipids for biodiesel and amino acids for cosmetic, antibiotic, and antiproliferative agents or bioremediation uses [6,7,8].

*Phaeodactylum tricornutum* is a well-recognized marine pennate diatom. It is a unicellular brown alga and the most-studied model diatom due to its nutrition-rich biochemical composition. It is widely used as feed for larvae because it produces compounds essential for the nutrition of other animals, such as proteins, carbohydrates, lipids, polyunsaturated fatty acids, and carotenoids (among them fucoxanthin) [5,9,10]. Moreover, it is characterized by fast growth and simple culturing requirements. Fucoxanthin is a yellowish carotenoid (xanthophyll) with an allenic carbon chain, an acyclic keto group, and a hydroxyl group at one of the β-ionone rings esterified with acetic acid [11,12]. This bioactive compound exhibits various biological properties beneficial to human health such as antiobesity, antidiabetic, anticancer, anti-allergic, and anti-inflammatory properties [13,14]. Furthermore, fucoxanthin content is much higher in diatoms than in seaweed (ranging from 0.22% to 2.17% of dry weight) [15].

The need for investigating novel alternative bioactive compounds extracted from natural biomass has increased. Green extraction techniques is a favored approach as it not only improves the extraction of high-value molecules but can also extract biomolecules without changing their conformation or bioactive properties and uses green solvents, thus contributing to environmental sustainability [16,17]. Several green extraction techniques exist, such as microwave-assisted extraction (MAE), ultrasound-assisted extraction (UAE), pulsed electric fields (PEF), pressurized liquid extraction (PLE), and supercritical fluids extraction (SFE) [16,18]. SFE has operational advantages over conventional extraction methods. SFE uses supercritical solvents such as carbon dioxide (CO_2_) due to their safety, low cost, and different polarity modifiers as co-solvent. Variables such as pressure, temperature, and solvent flow can be modulated in SFE to obtain different physicochemical properties of supercritical solvents such as density, diffusivity, viscosity, and dielectric constant [18,19].

“Algae-biorefinery” is a new concept describing the sustainable production of high-value molecules as co-products from algae biofuels as well as biofuels by the integration of bioprocessing and effective technologies in a cost-effective manner that minimizes environmental impact [20,21]. Biofuel is the result of energy conversion from natural biomass in biological processes that include anaerobic digestion (AD), alcoholic fermentation, photobiological hydrogen production, transesterification, and in situ transesterification [20,22]. Particularly, AD is focused on the production of biogas, primarily methane and carbon dioxide, with traces of other gases such as hydrogen sulfide by the conversion of organic material [23]. This process takes place in four sequential stages: hydrolysis, acidogenesis, acetogenesis, and methanogenesis [24,25].

Lipids or other compounds in value-added extraction can increase the biodegradability of microalgae; however, it depends on the solvent used in the extraction process. After extraction, methane production can be inhibited through AD [26]. Lage et al. [27] reported that the use of extracted microalgae increased the potential of methane production when the rest of the solvents were evaporated compared with the system where extracted solvent-impregnated microalgae were used. This situation could be explained by the negative effect of organic solvents [27]. Some organic solvents exhibit an inhibitory effect as chloroform, which disrupts the microbial cell membrane, thus compromising cell viability [28], or hexane and isopropanol [27]. Conversely, the results of the Cord-Ruwisch [29] study showed that ethanol and lactic acid were the intermediate products that could be potentially produced during the acid stage of anaerobic fermentation.

Evaporation of organic solvents in a previous stage of anaerobic fermentation is an alternative to improve the results of AD [26,27]. However, evaporation is an energy-demanding process that should be evaluated to determine the costs and the effect on the overall energy balance of the digestion process. The use of green technologies such as SFE avoids all the problems associated with the use of inhibitory organic solvents by facilitating the valorization of extracted microalgae through AD.

In this study, we performed supercritical carbon dioxide (SCO_2_) extraction of fucoxanthin from the diatom *P. tricornutum* and AD of the extracted microalga under microalgae biorefinery frame. We also studied the effects of factors such as temperature, pressure, and co-solvent concentration on variables such as the total extraction yield, fucoxanthin purity, and recovery. Finally, the biochemical composition of *P. tricornutum* and methane yields before and after the SFE process were studied to determine how the use of a green extraction system affects methane production.

## 2. Results and Discussion

### 2.1. Effects of Pressure and Temperature on the Yield of Fucoxanthin from P. tricornutum

**Figure 1:**
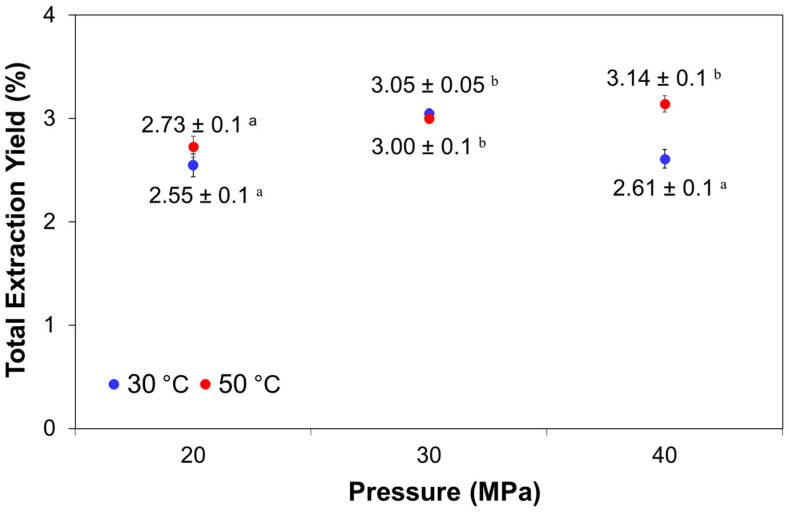
Total extraction yield obtained from *P. tricornutum* at different pressure and temperature conditions by supercritical fluid extraction. Different superscripted alphabets (a,b) indicate significant differences (*p* < 0.05).

Total extraction yields for each operating conditions (temperature of 30 °C and 50 °C and pressure of 20, 30, and 40 MPa) are summarized in Figure 1.

The results are expressed as the percentage of g extract per g dry weight of *P. tricornutum*, and the values were obtained at the end of extraction (60 min). This extraction time was established as optimal in previous studies on carotenoids from microalgae [30] and was considered profitable for minimizing its operational costs in the process. In general, total extraction yields were slightly affected by the combination of pressure and temperature factors. The highest total yields were without significant differences (*p* < 0.05) between them, if arranged from the highest to the lowest value, at the highest pressure and temperature (40 MPa at 50 °C, 3.14 ± 0.03%), followed by 30 MPa at 30 °C and 30 MPa at 50 °C (3.05 ± 0.05% and 3.00 ± 0.09%), respectively. On the other hand, the lowest extraction yields, which in turn did not have significant differences (*p* < 0.05) between them, were obtained at 20 MPa at 30 °C (2.55% *w/w*) together with the condition of 40 MPa at 30 °C and 20 MPa at 50 °C, which were 2.61 and 2.73% *w/w*, respectively (Figure 1).

As shown in Table 1 and the operating conditions, increasing the pressure at constant temperature increases the CO_2_ density from 0.890 to 0.988 g/mL for pressures of 20–40 MPa at 30 °C and from 0.784 to 0.923 g/mL for pressures of 20–40 MPa at 50 °C. Under these conditions of increased pressure, the total extraction yield (Figure 1) was slightly improved without significant differences (*p* < 0.05) for 30 MPa at 30 °C and 50 °C, as well as at 40 MPa at 50 °C, except for the conditions of 20 MPa at 30 °C and 50 °C and that of 40 MPa at 30 °C. In the pressure range of 30 to 40 MPa at 30 °C, a decrease in yield was observed, and it could be due to the volatility of the extracts under these SFE conditions, or the component solubility is lowered as the polarity and/or the molecular weights of the solutes are increased [19].

Table 1 also shows the purity, calculated as mg fucoxanthin extracted by SFE per g of total extract, and recovery, calculated as the percentage of the weight of fucoxanthin extracted by conventional extraction per grams of biomass as the reference weight of this pigment (equal to 9.82 ± 0.70 mg/g biomass) from of *P. tricornutum*. The different temperature and pressure conditions had a significant effect on these fucoxanthin variables, according to Tukey’s test. Particularly, the highest fucoxanthin purity and recovery were reached for a pressure of 30 MPa at 30 °C (60.62 ± 0.40 mg/g and 18.82 ± 0.12% *w/w*, respectively), being significant (*p* < 0.05) for the rest of the processes. Under these conditions, the density and superficial CO_2_ velocity were 0.948 g/mL and 0.401 mm/s, respectively, being the second-highest density and lowest superficial velocity, exceeded only by 30 °C and 40 MPa, whose values were 0.988 g/mL and 0.384 mm/s. However, in this treatment, the purity and recovery of fucoxanthin were not the highest. As shown in Table 1, the values of the superficial CO_2_ velocities were in the range of 0.384–0.484 mm/s, indicating slight variation, because the mass flow rate (3.62 g CO_2_/min) and the area of the extraction vessel (1589 cm^2^) were constant, the variations were only due to the density of CO_2_ that depends on temperature and pressure.

At low temperatures, the purity was higher than that at high temperatures under the same pressure values. This tendency was similar for fucoxanthin recoveries except for 40 MPa and 50 °C, which was approximately two-fold higher than that at 40 MPa and 30 °C. In contrast, at low pressure and under both temperature conditions, fucoxanthin purity and recovery were the lowest.

The particle size distribution is another important parameter in SFE as it directly affects the porosity and physical properties of the extraction bed. The particle diameter is inversely proportional to the total extraction yield [31,32]. On the other hand, a reduction in the final particle diameter increases the surface-to-volume ratio of the substrate [33]. The mean particle size of the *Phaeodactylum tricornutum* powder was D_P_ = 0.074 ± 0.004 mm, which is considered an ultrafine powder. Crampon et al. [34] reported that the smaller the particle, the faster the kinetics of the extraction, thus higher the yields. The disintegration of cells is essential in the recovery of intracellular products from algae.

The effect of temperature on extraction yield is more complex than that of pressure. This behavior is known as “crossover pressure” and is defined as the point where the vapor pressure of the compounds in the extract is more pronounced than the effect of solvent density over the solubility of the compounds [35,36]. The crossover region is a phenomenological observation that is supported by a relevant number of experimental studies. This phenomenon refers to the regions of temperature and pressure in the near-critical fluid state where the solute solubility decreases with an isobaric increase in temperature [37].

The effect of temperature on solubility is complex, and the crossover of the solubility isotherm occurs when pressure is considered as a variable. This phenomenon is affected by solvent density and solute vapor pressure. The vapor pressure increases when temperature and solubility increase; on the contrary, density and solvent power decrease. Consequently, solubility decreases because the density of the solvent is the dominant factor. After passing the crossover area, and when the temperature is increased, the vapor pressure of the solute becomes a dominant factor, and the solubility increases. However, this contrasting behavior is often attributed to solvent density and solute vapor pressure [38].

Foster et al. [39] reported that crossover pressure is a phenomenological observation that appears to reflect a characteristic of the solute–solvent system. Furthermore, it is also of fundamental significance in providing a direct indicator of the reliability and consistency of experimental solubility data. As a consequence, it may also be used to introduce a phenomenological constraint on thermodynamic models proposed to describe solute–supercritical fluid phase equilibria. This means that, at pressures lower than the crossover pressure, the effect of CO_2_ density is more relevant, whereas for pressures higher than the crossover pressure, the effect of vapor pressure at the determined temperature is more relevant for increasing the extraction yield. This is a common behavior for carotenoids obtained by SFE from vegetable matrices [37].

Fabrowska et al. [40] extracted carotenoids and phenolic compounds from the freshwater macroalgae *Cladophora glomerata* by optimization of the same parameters included in our study (temperature, pressure, and % ethanol as a co-solvent). The extraction yields were less than our yields without the use of extractants such as 0.6–0.9% *w/w*. The higher value of carotenoids recovery, represented with fucoxanthin as a majority, was 6.17 ± 0.24 mg/g of the extract under ~30 MPa and 50 °C, being similar conditions, except for the temperature. Ruiz-Domínguez et al. [41] used a Box–Behnken design with desirability function to determine the bioactive composition from the microalga *Isochrysis galbana* by SFE, and the parameters studied were pressure (20–40 MPa), temperature (40–60 °C), and co-solvent (0–8% ethanol) with a CO_2_ flow rate of 7.2 g/min for 120 min. The extraction yield without the presence of ethanol was in a similar range (from 1.09% to 2.28% *w/w*), and the highest was 2.28 ± 0.11% *w/w* under 40 MPa at 50 °C. They also determined total carotenoids recovery (included fucoxanthin) with an optimum value of 16.61 ± 0.74% at 40 MPa and 50 °C without co-solvent and 77.93 ± 2.87% at 30 MPa, 60 °C, and 8% *v/v* ethanol. Roh et al. [42] extracted fucoxanthin and polyphenols from the brown macroalga *Undaria pinnatifida* using SCO_2_ and ethanol as co-solvent. The highest yields of fucoxanthin in the presence of ethanol were at ~20 MPa at ~50 °C with a value of fucoxanthin recovery much lower than our results (approximately 0.00753 µg fucoxanthin per g freeze-dried sample). Therefore, our results showed that an increase in pressure (from 20 to 30 MPa) at a constant temperature (30 °C) increased the solvating power of carbon dioxide as supercritical fluids favoring fucoxanthin extraction. However, the temperature is a sensitive factor at high ranges, as it can degrade the targeted compound even though it can increase the solubility of this component [43].

Although all results of yield and recovery of fucoxanthin might be improved, the optimal conditions of pressure and temperature for the highest purity and recovery of fucoxanthin were 30 MPa and 30 °C and were selected for evaluating the behavior under ethanol as an extractant by SFE.

### 2.2. Effects of Ethanol as a Co-Solvent Factor on the Yield of Fucoxanthin from Phaeodactylum tricornutum

**Figure 2:**
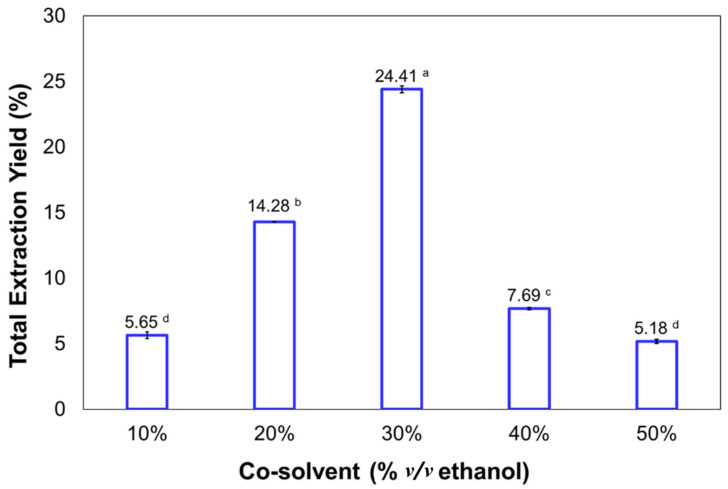
Total extraction yield obtained from *P. tricornutum* at different concentrations of co-solvent under 30 MPa and 30 °C by supercritical fluid extraction. Different superscripted alphabets (a–d) indicate significant differences (*p* < 0.05) and mean ± standard deviation (SD ≤ 5%, *n* = 3).

Many studies have reported that the use of ethanol as an extractant improves the yields of fucoxanthin due to its polarity and affinity with carotenoid molecules [44,45]. The optimal pressure and temperature for the highest purity and recovery of fucoxanthin along with the presence of co-solvent were used for evaluating the effect on the extraction process. Figure 2 shows the total extraction yield obtained under different concentrations of ethanol from 10% to 50% *v/v*. Here, the co-solvent improved the extraction yield, being at 30% *v/v* of ethanol, the highest value was obtained with 24.41% *w/w*, followed by 20% *v/v* of co-solvent with a value of 14.28% *w/w*. However, the lowest value was obtained at 10% and 50% *v/v* of ethanol, with 5.65% and 5.18% *w/w* of total extraction yields, respectively.

In general, the effect of co-solvent was significant; 30% *v/v* ethanol improved total yield to 8-fold higher than that using 30 MPa, 30 °C, and only carbon dioxide as a solvent. Many studies have reported that SFE is an excellent technique for the selective extraction of bioactive compounds such as carotenoids from various microalgae [16,43,46]. As a general rule, the extraction improves when SCO_2_ is used, whereas the presence of co-solvent or extractant such as ethanol enhances the efficiency of the supercritical process but disparages the selectivity [16].

On the other hand, Table 2 shows fucoxanthin purity and recovery under 30 MPa and 30 °C at different percentages of co-solvent. 

The highest values with no significant differences in fucoxanthin purity were 85.03 ± 7.67 mg/g and 74.73 ± 2.45 mg/g extract using 40 and 50% *v/v* of ethanol, respectively. However, the lowest purity was 13.75 ± 0.65 mg/g extract at 30% *v/v* of co-solvent despite the extraction yield being higher in this condition.

The highest fucoxanthin recovery with significant differences among others was 66.60 ± 6.00% *w/w* under 40% *v/v* of ethanol, whereas the lowest was 12.69 ± 1.14% *w/w* using 10% *v/v* of ethanol. The fucoxanthin purity and recovery reached the maximum value under the same conditions of 30 MPa, 30 °C, and 40% *v/v* ethanol. As reported by Gómez-Loredo et al. [47], an increasing concentration of co-solvent could induce a decrease in the purity and recovery of fucoxanthin as other compounds that were the part of the extract could transfer with this carotenoid. At times, up to a certain value, the addition of co-solvent has no further effect. This could happen at 50% *v/v* ethanol where fucoxanthin purity and recovery begin to decrease.

Extraction of fucoxanthin from different natural sources using conventional solvent, pressurized liquid extraction, or supercritical fluids extraction has been reported. For example, Conde et al. [48] showed ethanol concentration (0.5–10% *v/v*) as a significant factor in the supercritical CO_2_ extraction process from the macroalgae *Sargassum muticum*. The improvement in ethanol concentration resulted in total yields up to 3 times higher, radical scavenging capacity up to 2.5 times higher, and fucoxanthin extraction yield up to 90 times higher, thus reaching approximately 12 mg fucoxanthin/100 mg dry biomass under 20 MPa at 30 °C and 10% *v/v* ethanol. Gilbert-López et al. [49] compared MAE and PLE as green techniques for the extraction of bioactive compounds from *P. tricornutum*. The total yield (23.95% *w/w*), fucoxanthin purity, and its recovery (32.29 mg/g extract and 54.41% *w/w*, respectively) were the highest under 50 °C and 100% ethanol using PLE. Our results of fucoxanthin purity and recovery are higher than these results, although the *P. tricornutum* strain described by Gilbert-López et al. [49] reached up to 1.5-times higher concentration of fucoxanthin by conventional extraction than our results (maceration in acetone during 24 h at room temperature was around of 59.5 ± 4.7 mg/g extract). All results corroborated that *P. tricornutum* is an important source of bioactive compounds such as fucoxanthin and is better than other species such as macroalgae.

### 2.3. Biochemical Composition and the Effects of SFE on AD Using P. tricornutum

Table 3 presents the physicochemical characterization of the diatom *P. tricornutum* and supercritical-fluid-extracted *P. tricornutum.*

As shown, most of the analyzed parameters have maintained similar values in both groups. Only metal content, which increased in all cases after SFE, and the P_T_ –P_2_O_5_ parameter decreased by four times after SFE. It should be highlighted that the metal content reached non-toxic or inhibiting concentrations. The maintenance of nutrients such as nitrogen (N-TKN) or phosphorous (P_T_ –P_2_O_5_) in the diatom *P. tricornutum* is essential for the microorganisms responsible for performing AD.

The comparison of the biochemical composition of *P. tricornutum* biomass and other strains is presented in Table 4. Biogas production from microalgae depends on their biochemical composition and other factors such as operating conditions, cell wall nature, C/N ratios and ammonia release, and sodium in marine species [50,51,52]. The analysis of the biochemical composition may help in the comprehension of the methane yields obtained. The best theoretical methane yields obtained are for lipids, followed by proteins and carbohydrates, with values 1.014 L CH_4_/g VS, 0.851 L CH_4_/g VS, and 0.415 L CH_4_/g VS, respectively [51]. These values could be accurate considering the composition of the specific microalga species. 

Generally, the exhausted diatom has the lowest macronutrient content due to the extraction process, highlighting the decrease in lipid and protein content by more than half. The biochemical composition of pretreated *P. tricornutum* markedly decreased with the extraction of 68.99% and 60.19% of lipids and proteins, respectively, until the values of 5.01 ± 0.01% and 14.41 ± 0.62% *w/w*, respectively, were obtained. However, only 29.15% of carbohydrates were extracted. On the other hand, the protein content differed significantly (*p* ≤ 0.05) among the strains, followed by lipid and carbohydrate contents. The protein content ranged from 38.8 ± 0.11% to 26.95 ± 0.05% *w/w* (excluding *P. tricornutum* after SFE) with *P. tricornutum* F&M-M4 showing the highest value (Table 4). For carbohydrate content, all strains showed similar values with slight differences. Particularly, the carbohydrate content of *P. tricornutum* in our study was higher (18.80 ± 1.12% *w/w*) than the *P. tricornutum* carbohydrate content (16.91 ± 1.61% *w/w*) reported by Di Lena et al. [53]. *P. tricornutum* F&M-M4 had the highest lipid content, followed by a diatom described by Bernaerts et al. [54] (17.1 ± 0.9% *w/w*). In the case of extracted *P. tricornutum,* the lipid content was significantly decreased compared with the initial content in the biomass before extraction because especially the lipid fraction was recovered through supercritical CO_2_-extraction technology.

Globally, such results have confirmed *P. tricornutum* as complete and nutritive biomass; hence, it is important in aquaculture feeds and food applications [5,9,10,55]. Nevertheless, the biochemical compositions of *P. tricornutum* found in previous studies are variable and highly dependent on strains, culturing conditions, and growth stages [54,56].

As shown in Table 4, our supercritical-fluid-extracted *P. tricornutum* or exhausted biomass was rich in proteins, carbohydrates, and to a lesser extent in lipids. This could be used in different integrated processes in algae biorefinery. Hence, in our study, the production of bioenergy in the form of biogas was evaluated.

### 2.4. BMP of Diatom P. tricornutum and Extracted P. tricornutum by SFE

Typically, the yield of biogas obtained by AD is associated with the biochemical composition and VS content of the biomass used as a substrate. Particularly, the AD of microalgae can be affected by different factors such as low C/N ratio, thick cell walls banking on the microalgal genus, and high concentrations of sodium for marine species [50,52,58].

*P. tricornutum* is the only species in the genus *Phaeodactylum*, which is rich in bioactive compounds such as fucoxanthin and fatty acids [10,55]. However, this genus has a silicious wall, one of the main drawbacks when considering the microalgae for AD that could affect the results of this process. When intracellular compounds of interest such as fucoxanthin are extracted by SFE from microalgae, the action of temperature, pressure, and the use of ethanol as a co-solvent can be considered as a pretreatment. SFE helps in the extraction of fucoxanthin, but the breakage of cell structures also favors the release of other compounds, which facilitates the action of microorganisms that perform the hydrolytic steps of AD. After SFE, the cell wall and structure breaks to some extent, and the remaining compounds and cell material are released into the surrounding environment, which can be used as substrates to feed anaerobic microorganisms [59].

**Figure 3:**
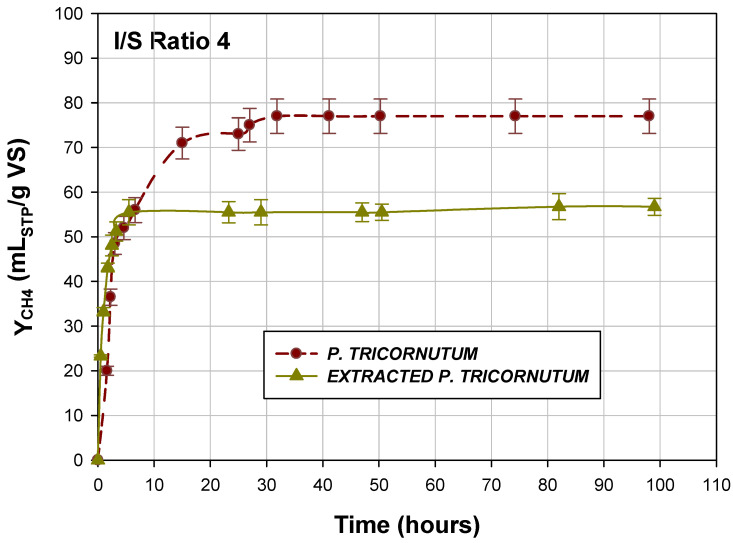
Methane yields obtained for diatom *P. tricornutum* and supercritical-fluid-extracted microalga *P. tricornutum*.

The methane yields obtained for the whole *P. tricornutum* and the extracted diatom were 77.2 ± 3.85 LSTP CH_4_/kg VS and 56.7 ± 1.90 LSTP CH_4_/kg VS, respectively (Figure 3). 

In both cases, a methane yield of around 55 LSTP CH_4_/kg VS was obtained in less than 10 h, highlighting the fast biodegradability of the substrate and microbial activity at the beginning of AD. Caporgno et al. [60] obtained methane yields of 257 ± 8 mL of CH_4_/g VS and 180 ± 6 mL of CH_4_/g VS, respectively, for *P. tricornutum* after lipid extraction for biodiesel production with traditional solvents such as hexane and methanol/hexane mixture. Zhao et al. [26] obtained the yield of 339 ± 13 mL of CH_4_/g VS for the lipid-extracted microalgal biomass using hexane and evaporating it after the extraction at 105 °C in a hood. Studies on the AD of *P. tricornutum* are limited, and the methane yields reported by Caporgno et al. [60], Zhao et al. [26], Frigon et al. [61], or Zamalloa et al. [62] could not be compared with the yields obtained in the present study because the extraction methods were not the same, nor did the extracted compounds include fucoxanthin, and the extraction of different compounds can affect AD methane yields differently.

In addition, a high or low concentration of a co-solvent (as a remainder) at the end of the extraction process of added high-value products can increase methane production when they are biodegradable. In our study, the methane yield obtained for the whole diatom was higher (77.2 ± 3.85 LSTP CH_4_/kg VS) than that obtained for the extracted diatom (56.7 ± 1.90 LSTP CH_4_/kg VS), resulting in a 26.6% reduction in the methane yield obtained from the whole microorganism. However, from the environmental and economic point of view, through the AD of the extracted *P. tricornutum,* biogas was obtained, which could reduce the energy costs for fucoxanthin extraction by SFE and/or contribute to the reduction in the costs of growing new microalgal biomass conferring the circularity to the process.

### 2.5. Characteristics of Effluents after AD

Table 5 shows the characterization of the effluents or digests obtained after BMP assays of the microalgae *P. tricornutum* and the supercritical-fluid-extracted *P. tricornutum*. Conductivity was stable in both microalgae, with values around 17.75 to 18.14 mS/cm in all effluents obtained and loads assayed. The pH in these effluents increased with the second load in the BMP assays of both diatoms. The pH values obtained in the second load of the supercritical-fluid-extracted *P. tricornutum* were 9.05 ± 0.02, which are especially high for an anaerobic system, for which the optimal pH range is from 6.7 to 7.5, close to neutral ranges. In anaerobic processes and at this pH, VFA and ammonia almost dissociate. 

**Figure 4:**
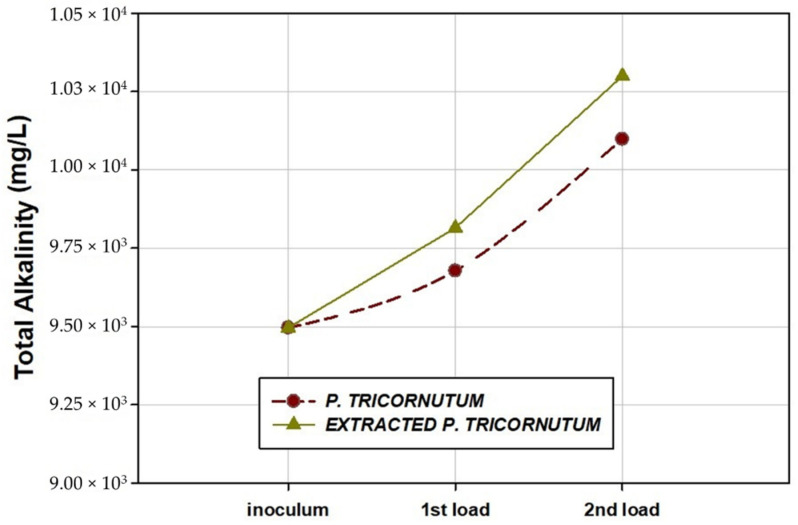
Total alkalinity of the effluents obtained after biochemical methane potential assays (first load and second load) for the diatom *P. tricornutum* and supercritical-fluid-extracted *P. tricornutum*.

Figure 4 shows the values of T-Alk measured in the effluents obtained after the BMP assays of diatom *P. tricornutum* and supercritical-fluid-extracted *P. tricornutum*.

T-Alk is an important parameter for AD systems. T-Alk gives anaerobic systems the capacity to cope with pH fluctuations. Most anaerobic reactors have T-Alks between 1500 and 5000 mg CaCO_3_/L, although values above 5000 mg CaCO_3_/L found in some reactors exhibited a slightly negative effect on the process [63]. In the present study, T-Alk values of the effluents were high, 9678 ± 90 mg CaCO_3_/L and 10,098 ± 105 mg CaCO_3_/L for the first and the second loads assayed with *P. tricornutum* and 9815 ± 115 and 10,300 ± 95 mg CaCO_3_/L for the first and second loads with supercritical-fluid-extracted *P. tricornutum*, respectively. This increase in T-Alk could be responsible for the increase in ammonia concentration. The initial growth of ammonia in the media can increase buffer capacity [64]; however, the high concentrations of ammonia can inhibit methanogenesis. 

**Figure 5:**
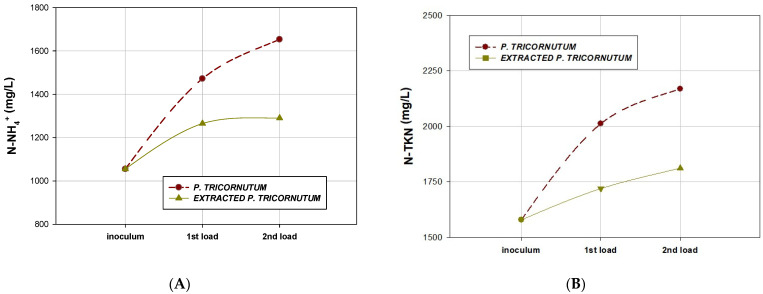
Ammoniacal nitrogen (**A**) and total Kjeldahl nitrogen (**B**) content in the effluents obtained after biochemical methane potential assays (first load and second load) for the diatom *P. tricornutum* and supercritical-fluid-extracted *P. tricornutum*.

In anaerobic digesters, ammonium and free ammonia are in equilibrium, and free ammonia, a form of nitrogen, is more dangerous for these systems. Free ammonia concentrations are usually higher at higher pH and temperatures, reaching an inhibitory concentration of 150 mg N–NH_3_/L [65]. In the present study, N–NH_4_^+^ concentration increased from 1472 ± 60 to 1653 ± 50 mg/L when moving from the first load to the second load in the reactors digesting *P. tricornutum*. On the other hand, N–NH_4_^+^ concentrations were almost constant for the first and second loads of the extracted *P. tricornutum*, with values of 1265 ± 5 and 1290 ± 10 mg/L for the first and second load assayed, respectively (Figure 5A). 

The values obtained for the whole diatom *P. tricornutum* were high and close to inhibitory values obtained in anaerobic environments, between 1500 and 3000 mg N–NH_4_^+^/L [66]. Inhibitory concentration values can be higher in an adapted process; some studies have shown the tolerance of microalgae to ammonia concentrations between 1500–7000 mg N–NH_4_^+^/L [67]. Factors such as the origin of the inoculum, temperature, and types of substrates may affect this wide range of inhibitory concentrations of N–NH_4_^+^ [65]. 

The N-TKN (Figure 5B) present in the effluents obtained after the first and second load of the diatom *P. tricornutum* were higher than that obtained after the first and second load of the supercritical-fluid-extracted *P. tricornutum,* obtaining values of 2013 ± 25 mg/L and 2169 ± 25 mg/L for the first and second loads of the whole *P. tricornutum*, respectively. These values were lower for the extracted *P. tricornutum*, 1720 ± 10 and 1812 ± 15 mg/L for the first and second loads, respectively. These values could be responsible for a 39.8% reduction in proteins, which decreased from 36.20% to 14.41% (*w/w*) for the diatom *P. tricornutum* due to SFE, as described in Section 2.3 and Table 4.

**Figure 6:**
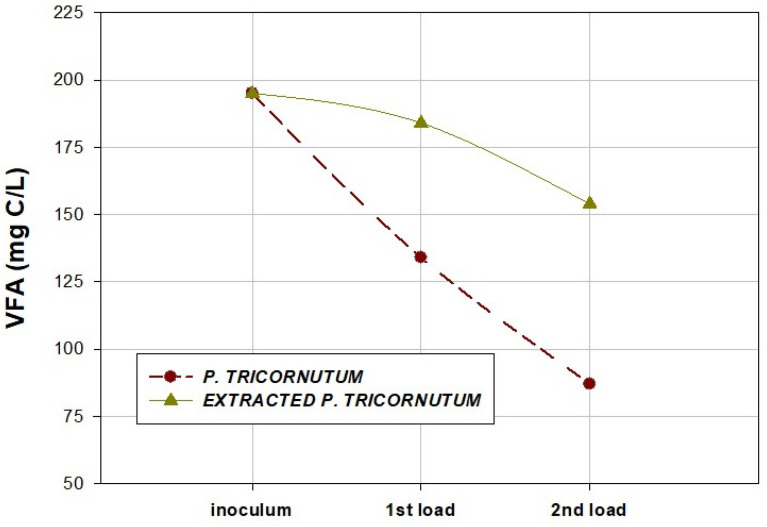
Concentrations of volatile fatty acids (VFA) obtained in the effluents after biochemical methane potential (BMP) assays (first load and second load) for the diatom *P. tricornutum* and supercritical-fluid-extracted *P. tricornutum*.

Figure 6 shows the VFA evolution in each load studied for the diatom *P. tricornutum* and supercritical-fluid-extracted *P. tricornutum.* VFAs in the effluents after the BMP assays showed a decreasing trend at the end of the second load, decreasing values from 195 ± 5 mg C/L to 87 ± 5 mg C/L for the non-extracted diatom and from 190 ± 10 mg C/L to 154 ± 5 mg C/L for the supercritical-fluid-extracted *P. tricornutum*. The decrease in acidity was an indicator of the presence of an adequate amount of methanogenic bacteria.

Finally, it seems that metal content provided by the own diatom does not apparently affect the AD process. The use of microalgae (including diatoms) in co-digestion with rich carbon substrates is common for obtaining adequate nutrients and metal balance for the anaerobic system [68].

## 3. Materials and Methods

### 3.1. Biomass and Chemicals

The diatom *Phaeodactylum tricornutum* (CIB-41 from CIBNOR (https://www.cibnor.gob.mx/, accessed on 22 November 2021) La Paz, Mexico) was obtained from the Mexican Company “Microalgas Oleas de México S.A.” (Guadalajara, Mexico). The diatom was cultured in F/2 culture medium as reported previously by Guillard [69] and harvested at the end of the exponential phase. The microalga (diatom) was provided freeze-dried (freeze-dry system Labconco Freezone 18 L Benchtop Freeze Dry System, Kansas, MO, USA), packed in vacuum-sealed plastic bags and then was stored at 4 ± 2 °C in the dark until use. All solutions used in this study were of analytical grade except for the standard solutions and chromatographic solvents. For supercritical fluid extraction, carbon dioxide and ethanol were purchased from Indura Air Products Group (Santiago, Chile) and Sigma-Aldrich (Santiago, Chile), respectively. Bovine Serum Albumin (BSA) (as the standard in protein analysis) and glucose (as the standard in carbohydrate determination) were acquired from Sigma-Aldrich (Santiago, Chile). For fucoxanthin identification and quantification, a standard of >99% purity (Sigma-Aldrich, Santiago, Chile) was used. Ethyl acetate, acetonitrile, and ultrapure (MilliQ) water were of chromatographic grade (Sigma-Aldrich, Santiago, Chile) for high-performance liquid chromatography (HPLC).

### 3.2. Determination of Particle Size Distribution of the P. tricornutum Freeze-Dried Biomass 

The particle size distribution of lyophilized diatom powder was determined using an electromagnetic sieve shaker (CISA Sieving Technologies, BA200N model, Barcelona, Spain). Eight sieves with ISO 3310–1 (ASTM E11) sizes of 10, 12, 16, 18, 35, 60, 120, and 230 were used. After 15 min shaking, the material retained in each sieve and the bottom pan was weighed. The mean particle diameter (Dp) of the *P. tricornutum* powder was determined according to ASABE [70] using Equation (1).

(1) $$D_{P}=\exp\left\{\frac{\sum_{i=1}^{n}\left[\left.\sqrt{W_{i}\cdot \log\left(d_{i}\cdot d_{i+1}\right)}\right]\right.}{\sum_{i=1}^{n}W_{i}}\right\}$$

where D_P_ is the mean particle diameter (mm); d_i_ is the diameter of the sieve opening i (mm); d_i+1_ is the diameter of the sieve opening above sieve i (mm); W_i_ is the retained mass (g); and n is the total number of fractions. The samples were kept in sealed plastic bags in a refrigerator till further analysis.

### 3.3. SFE

The SFE experiments were performed using a commercial SFE unit (Spe-ed SFE Helix, Applied Separations, Allentown, PA, USA), which was described in detail by Salinas et al. [71]. The aim of this experiment was to evaluate the effect of extraction conditions such as the temperature of 30 °C and 50 °C; the pressure of 20, 30, and 40 MPa; and the concentration of ethanol as a co-solvent from 10% to 50% *v/v* on the extraction yield, fucoxanthin purity, and fucoxanthin recovery from the *P. tricornutum* biomass. Initially, different temperature and pressure conditions were evaluated using the freeze-dried biomass to optimize temperature and pressure parameters. For each extraction assay, 2.0 g of *P. tricornutum* biomass was packaged in a 24 mL extraction vessel (nominal volume), with the dimensions of 0.56 inch (1.4224 cm) of internal diameter and 5.9 inches (14.986 cm) of internal height (ASI Part Number 6414/7975; Applied Separations, Allentown, PA, USA), occupying a volume of 2.54 mL, resulting in a bed density of 0.787 g/mL, which is approximately 10.6% of the total volume of the extraction vessel. The empty space of the extraction vessel was filled with 1.0 mm glass beads. The extraction vessel containing the biomass was assembled into the heated jacket at a selected temperature and kept for 15 min until the desired temperature was stabilized. CO_2_ was pumped until the desired pressure was reached, and the system was maintained for 10 min (static time) and the total time of extraction was set at 60 min according to a previous report on the extraction of carotenoids (xanthophyll) from microalgae [30]. The CO_2_ flow rate was measured using an electronic flow meter installed at the exit of the sample collection flask. After the static time, the outlet valve of the bed was opened, and the micrometric valve was adjusted to reach a CO_2_ flow rate of 2 L/min that corresponds to a mass flow rate of 3.62 g/min. At this mass flow rate, superficial CO_2_ velocities (U: mm/s) through the extraction vessel were calculated according to the temperature (30 °C and 50 °C) and pressure (20, 30, and 40 MPa) of each treatment. 

A factor that critically affects the cost of SFE processes for solid substrates is the extraction time [72], which in turn affects extraction yield that depends on substrate pretreatment, particle size (Dp), CO_2_ temperature (T), pressure (P), and superficial velocity (U) [73]. This flow of CO_2_ during 60 min corresponds to a ratio of solvent to feed (S/F) of 108.6. The effect of operative conditions on the yield and extraction of fucoxanthin was expressed in terms of the total yield of extract and fucoxanthin purity and recovery from *P. tricornutum*, respectively. These variables were calculated using the following Equations (2)–(4):

(2) $$\mathrm{Total}\;\mathrm{Yield}\;(\%)=\left(We/Wb\right)\times 100$$

(3) Purity (mg/g)=Wc/We

(4) $$\mathrm{Recovery}\;(\%)=\left(Wc/Wt\right)\times 100$$

where *We* is the weight of the extract (g), *Wb* is the weight of biomass (g); *Wc* is the weight of the fucoxanthin extracted (mg), and *Wt* is the reference weight of the fucoxanthin from conventional extraction (mg). This value was calculated on the basis of the initial content of fucoxanthin pigment in the diatom, expressed as mg of extract per gram of dry weight of *P. tricornutum* (equals to 9.82 ± 0.70 mg/g). Each experimental condition was replicated three to five times, and standard deviation (SD) was calculated for each value.

### 3.4. Fucoxanthin Quantification by HPLC

Fucoxanthin was extracted from 50 mg of supercritical fluids extracts, and methanol (0.05% *w/v* BHT) was added in all samples. The aliquots were filtered (Ø = 0.22 µm filter) and transferred into an amber vial for chromatographic analysis. Subsequently, fucoxanthin was separated and identified using the HPLC system, model 7100 (Merck Hitachi LaChrom, Tokyo, Japan) equipped with three pumps (flow rate of 1 mL/min), and a UV-Vis detector was used at 450 nm. The samples (20 µL) were injected into a capillary column (C18, 250 mm × 4.6 mm, 5 µm, Restek, Bellefonte, PA, USA). The mobile phase consisted of solvent A (ethyl acetate) and solvent B (acetonitrile:water 90:10). The gradient procedure was 100% solvent B for 10 min; 50% solvent B for 4 min; 40% solvent B for 2 min; and 100% solvent B for 1 min. Fucoxanthin was identified by comparing the retention times with that of the reference standard. Fresh standard solutions (1ppm to 20 ppm of fucoxanthin) were prepared and injected to generate the standard fucoxanthin curve. The calculation of the free fucoxanthin concentration in the samples was performed using the standard curve that was found to be linear over the required range (1–25 µg/mL; *R*^2^ = 0.9971). All analyses were performed in triplicate.

### 3.5. Characterization of Biochemical Composition 

The characterization of biochemical composition was performed from the original biomass, the whole *P. tricornutum*, and the exhausted biomass. The exhausted biomass was a unique sample composed of a mixture resulting from the biomass pool after all SFE extractions studied. The protein content of *P. tricornutum* was analyzed colorimetrically using a microplate reader (Synergy HTX Multi-Mode microplate reader, software Gen5 2.0, BioTek Instruments, Winooski, VT, USA) and BSA solutions for the standard curve (0.0−2.0 mg/mL) by following the Lowry method [74]. The results are expressed as the percentage of total proteins with respect to their dry biomass (% *w/w*). Total carbohydrate content was determined according to the modified protocols of Dubois et al. [75] and Geresh et al. [76] using a series of glucose solutions as the standard (0.0−1.0 mg/mL). The biomass used for carbohydrate measurement was subjected to acid hydrolysis for cell rupture. Briefly, 20 mg of the dry biomass was incubated in 5 mL of 2.5 M HCl for 3 h at 100 °C (Spectroquant Thermoreactor TR 320, Merck, Darmstadt, Germany). This mixture was neutralized with 5 mL of 2.5 M NaOH to obtain the microalgal extract. Finally, aliquots of 278 µL of extract, 167 µL of phenol solution (5% *w/v*), and 1000 µL of concentrated sulfuric acid were incubated for 30 min at room temperature, and the absorbance of these samples was measured at 483 nm using the microplate reader. The results are expressed as the percentage of total carbohydrates with respect to the dry biomass (% *w/w*). Total lipids were obtained according to the method of Axelsson et al. [77]. The dry biomass of *P. tricornutum* (~20 mg) was resuspended in chloroform:methanol (2:1, *v/v*) by manually shaking the tube vigorously for a few seconds or until the biomass was dispersed in the solvent system. Finally, 0.73% (*w/v*) NaCl water solution was added to produce a 2:1:0.8 system of chloroform:methanol:water (*v/v/v*). The previous evaporation with N_2_ was performed using Flexivap Workstation (Glas-Col 109A YH-1, Terre Haute, IN, USA). Lipid content was expressed as the percentage of total lipids with respect to the biomass (% *w/w*). Proteins, total carbohydrates, and total lipids are expressed as the average of a triplicate (n = 3), and the data are expressed as mean ± SD.

### 3.6. Analytical Methods

The parameters pH, conductivity, total alkalinity (T-Alk), total solids, volatile solids (VS), mineral solids, total chemical oxygen demand (TCOD), soluble chemical oxygen demand, and volatile fatty acids (VFA) were determined according to the Standard Methods of the APHA et al. [78]. A Shimadzu model TOC-VCSH carbon analyzer was used for the determination of total organic carbon by the catalytic oxidation of total carbon present in the sample to CO_2_ and detection by infrared spectrophotometry. Metal content was determined by flame photometry [78] with a Perkin-Elmer A Analyst 300 atomic absorption spectrophotometer. The methodology proposed by the US Department of Agriculture and the US Composting Council [79] was used for the determination of ammoniacal nitrogen (N-NH_4_^+^), total Kjeldahl nitrogen (N-TKN), and phosphorus (P-P_2_O_5_) content. Volatile fatty acids (VFA) such as acetic, propionic, butyric, isobutyric, valeric, and isovaleric acids were determined using a Hewlett-Packard HP-5890 gas chromatograph equipped with a 15 m × 5.3 × 10^−4^ m (i.d.) Nukol-silica semi-capillary column and a flame ionization detector.

### 3.7. Anaerobic Inoculum and BMP

Table 6 summarizes the main characteristics of the inoculum used for the biochemical methane potential (BMP) tests. The inoculum used was a baker’s yeast vinasse obtained from La Golondrina plant, the municipal water company (EMACSA), Córdoba, Spain.

The AD of *P. tricornutum* was assessed by performing BMP assays in batches. The 1.2 L reactors or digesters were maintained at mesophilic temperature (35 ± 2 °C) using a thermostatic water bath (LAUDA RTM 20) and were continuously agitated at 200 rpm. An inoculum-to-substrate ratio was maintained at 4 (on a VS basis). A total of 5.7 g inoculum of VS was added in each reactor using an effective volume of 1000 mL. The headspace of each flask was flushed with nitrogen to displace O_2_ and promote AD conditions. The CH_4_ produced was measured using a 1 L Boyle–Mariotte reservoir connected to each digester. NaOH solution (6N) was used assuming that the non-captured gas from biogas was methane. The volume of CH_4_ was measured by water displacement. All methane volumes and yields were measured under standard temperature and pressure conditions (STP): 0 °C and 1 atm. 

Before the experiments, the biomass was activated with GAL solution, a mixture of glucose (50 g/L), lactic acid (21 mL/L), and sodium acetate (25 g/L) with a TCOD concentration of 86,900 ± 75 mg O_2_/L and a pH of 7.05 ± 0.01. The experiments were performed in duplicate. Subsequently, the two digesters were fed with diatom *P. tricornutum* and the other two digesters with the extracted *P. tricornutum* (after the-SFE) to obtain CH_4_ yields from both substrates. 

### 3.8. Statistical Analysis

The Statgraphics Centurion XVIII^®^ Statistics software (StatPoint Technologies, Inc., Warrenton, VA, USA) was used for data elaboration and statistical analysis using a significance level of 95%. One-way analysis of variance, together with Tukey’s test, was used for group extracts based on statistically significant differences. The effect of each factor and its statistical significance for each response variable were analyzed. A p-value of <0.05 was considered statistically significant. All experiments were performed in triplicate (*n* = 3), and the data are expressed as the mean ± SD.

## 4. Conclusions

In this study, we evaluated the use of *P. tricornutum* as biomass in the algae biorefinery concept using SFE, a green method, and biogas production to close the loop in a circular schedule. Fucoxanthin was the first valuable molecule to be recovered due to its many possible health benefits and high food market value. The best operating SFE parameters for the extraction yield, purity, and recovery of fucoxanthin from *P. tricornutum* by SFE were assessed. These optimal conditions were 30 MPa pressure at 30 °C and 30% *v/v* ethanol for extraction yield and 30 MPa pressure at 30 °C and 40% *v/v* of the extractant for fucoxanthin purity and recovery. Fucoxanthin recovery improved by 3.5 times at 30 MPa and 30 °C in the presence of ethanol, the most significant variable of SFE. Moreover, the diatom *P. tricornutum* was confirmed to be a potentially valuable natural resource for nutritional or nutraceutical purposes due to its good biochemical composition. The use of *P. tricornutum* and extracted diatom for AD enhanced the benefit of obtaining bioenergy in the form of biogas, obtaining methane yields of 77.15 ± 3.85 LSTP CH4/kg VS and 56.66 ± 1.90 LSTP CH4/kg VS for the whole diatom and the extracted *P. tricornutum*, respectively, in addition to fucoxanthin extraction; thus, implying its use a circular economy strategy, minimizing the potential costs associated with SFE as a costly pretreatment process for biogas production.

Based on our findings, *P. tricornutum* can be a useful candidate to diversify its multipurpose use from biotechnological and bioactive applications to sustainable energy production, playing a key role in the “algae biorefinery” concept.

## Figures and Tables

**Table 1:** Fucoxanthin purity and recovery of extracts from *P. tricornutum* biomass obtained by supercritical fluid extraction under different extraction conditions.

SFE Conditions		CO_2_		Fucoxanthin	
P (MPa)	T (°C)	Density (g/mL)	Superficial Velocity (mm/s)	Purity (mg/g Extract)	Recovery (% *w/w*)
20	30	0.890	0.426	11.07 ± 0.46 ^a^	2.87 ± 0.12 ^a^
20	50	0.784	0.484	8.56 ± 0.14 ^a^	2.38 ± 0.04 ^a^
30	30	0.948	0.401	60.62 ± 0.40 ^d^	18.82 ± 0.12 ^e^
30	50	0.870	0.436	37.89 ± 1.00 ^c^	11.59 ± 0.31 ^d^
40	30	0.988	0.384	29.06 ± 1.63 ^b^	4.76 ± 0.19 ^b^
40	50	0.923	0.411	26.67 ± 2.08 ^b^	8.53 ± 0.66 ^c^

Acronyms: SFE (supercritical fluid extraction); P (pressure); and T (temperature). Different superscripted alphabets (a–e) indicate significant differences (*p* < 0.05) and the data are expressed as mean ± standard deviation (*n* = 3, SD ≤ 5%).

**Table 2:** The purity and recovery of fucoxanthin from the *P. tricornutum* biomass obtained by supercritical fluid extraction under 30 MPa and 30 °C at different concentrations of co-solvent conditions.

Co-Solvent (Ethanol, % *v/v*)	Ethanol + CO_2_ (g/min)	Fucoxanthin Purity (mg/g Extract)	Fucoxanthin Recovery (% *w/w*)
10	3.406	22.06 ± 1.92 ^ab^	12.69 ± 1.14 ^a^
20	3.208	28.65 ± 0.49 ^b^	41.68 ± 0.71 ^b^
30	3.002	13.75 ± 0.65 ^a^	34.17 ± 1.61 ^b^
40	2.801	85.03 ± 7.67 ^c^	66.60 ± 6.00 ^c^
50	2.591	74.73 ± 2.45 ^c^	39.46 ± 1.29 ^b^

Different superscripted alphabets (a–c) indicate significant differences (*p* < 0.05) and the data are expressed as mean ± standard deviation (*n* = 3, SD ≤ 5%).

**Table 3:** Characteristics of *P. tricornutum* before and after supercritical fluid extraction.

Parameters	*P. tricornutum*	Supercritical Fluid Extracted *P. tricornutum*
TS (mg/kg)	891,635 ± 1485	920,030 ± 1025
MS (mg/kg)	182,375 ± 2030	200,420 ± 4005
VS (mg/kg)	709,260 ± 3520	719,610 ± 1090
VS (%)	79.55	78.22
Humidity (%)	10.84 ± 0.07	8.00 ± 0.05
N-TKN (mg/kg)	56,167 ± 173	42,937 ± 4687
P_T_ –P_2_O_5_ (mg/g)	1.692 ± 0.038	0.433 ± 0.004
Cr (mg/kg)	10.9 ± 0.25	16.36 ± 0.10
Cd (mg/kg)	2.05 ± 0.30	4.26 ± 0.65
Pb (mg/kg)	3.25 ± 0.50	5.11 ±0.70
Ni (mg/kg)	4.11 ± 0.15	6.48 ± 0.68
Zn (mg/kg)	180.30 ± 0.85	255.43 ± 0.68
Cu (mg/kg)	12.95 ± 0.17	18.84 ± 0.08

Acronyms: TS: total solids, MS: mineral solids, VS: volatile solids, N-TKN: total Kjeldahl nitrogen, P_T_ –P_2_O_5_: total phosphorous.

**Table 4:** Biochemical composition of the diatom *P. tricornutum* before and after supercritical fluid extraction compared with the composition reported in previous studies.

Strain/Diatom	Proteins (% *w/w*)	Carbohydrates (% *w/w*)	Lipids (% *w/w*)	References
*P. tricornutum*	36.20 ± 1.20 ^d^	18.80 ± 1.12 ^b^	16.15 ± 0.75 ^c^	In this study
Supercritical fluid extracted *P. tricornutum*	14.41 ± 0.62 ^a^	13.32 ± 0.45 ^a^	5.01 ± 0.01 ^a^	In this study
*P. tricornutum* F&M-M4	38.8 ± 0.11 ^e^	11.0 ± 0.70 ^a^	20.5 ± 0.54 ^d^	[57]
*P. tricornutum*	29.4 ± 0.4 ^c^	10.5 ± 0.26 *^a^	17.1 ± 0.9 ^c^	[54]
*P. tricornutum*	26.95 ± 0.05 ^b^	16.91 ± 1.61^b^	12.73 ± 0.13 ^b^	[53]

* Sum of storage polysaccharides (SPS) and cell wall polysaccharides (CWPS). Note: Different superscripted alphabets (a–e) indicate significant differences (*p* < 0.05) and the data are expressed as mean ± standard deviation (*n* = 3, SD ≤ 5%). Nucleic acids and ash account for the percentage left to make 100% wt of proximal composition.

**Table 5:** Characterization of the effluents or digestates obtained after BMP assays on the diatom *P. tricornutum* and the supercritical fluid extracted *P. tricornutum*.

Variables	1st Load *P. Tricornutum*	2nd Load *P. Tricornutum*	1st Load Supercritical Fluid Extracted *P. Tricornutum*	2nd Load Supercritical Fluid Extracted *P. Tricornutum*
pH	8.26 ± 0.01	9.05 ± 0.02	8.00 ± 0.02	8.29 ± 0.01
Conductivity (mS/cm)	18.09 ± 0.02	18.14 ± 0.03	17.89 ± 0.01	17.75 ± 0.01
T-Alk (mg CO_3_Ca/L)	9678 ± 90	10,098 ± 105	9815 ± 115	10,300 ± 95
VFA (mg C/L)	134 ± 10	87 ± 5	184 ± 8	154 ± 5
VFA (mgCH_3_COOH/L)	335 ± 15	218 ± 13	525 ± 20	385 ± 13
TS (mg/L)	17,952 ± 435	18,606 ± 105	17,900 ± 80	18,325 ± 160
MS (mg/L)	11,177 ± 210	11,311 ± 85	11,940 ± 250	11,898 ± 350
VS (mg/L)	6775 ± 225	7295± 40	6260 ± 100	6620 ± 430
N-NH_4_^+^ (mg/L)	1472 ± 60	1653± 50	1265 ± 5	1290 ± 10
TKN (mg/L)	2013 ± 25	2169 ± 25	1720 ± 10	1812 ± 15
P_T_ –P_2_O_5_ (mg/L)	4.72 ± 0.02	5.56 ± 0.02	5.01 ± 0.02	5.83 ± 0.02
Cu (mg/L)	0.28 ± 0.01	0.17 ± 0.02	0.10 ± 0.01	0.05 ± 0.01
Ni (mg/L)	0.90 ± 0.01	0.22 ± 0.01	0.02 ± 0.01	<DL (0.05 mg/L)
Zn (mg/L)	0.98 ± 0.02	0.26 ± 0.03	0.30 ± 0.01	<DL (0.05 mg/L)
Pb (mg/L)	6.36 ± 0.06	5.46 ± 0.05	0.43 ± 0.01	0.21 ± 0.01
Cr (mg/L)	0.078 ± 0.002	<DL (0.05 mg/L)	2.96 ± 0.01	<DL (0.05 mg/L)
Cd (mg/L)	0.60 ± 0.01	0.22 ± 0.02	0.41 ± 0.01	<DL (0.05 mg/L)
Acetic acid (mg/L)	26.80 ± 2.56	28.4 ± 4.21	51.80± 5.20	27.95 ± 4.12
Propionic acid (mg/L)	6.12 ± 0.25	7.69 ± 2.10	14.48 ± 2.35	7.23 ± 1.98
Isobutiric acid (mg/L)	25.00 ± 1.00	27.54 ± 1.36	21.67 ± 1.38	4.87 ± 5.24
Butiric acid (mg/L)	8.35 ± 1.40	7.63 ± 1.02	25.89 ± 3.33	14.23 ± 3.07
Isovaleric acid (mg/L)	18.25 ± 3.15	25.08 ± 3.00	<DL (0.5 mg/L)	<DL (0.5 mg/L)
Valeric acid (mg/L)	<DL (0.5 mg/L)	<DL (0.5 mg/L)	<DL (0.5 mg/L)	<DL (0.5 mg/L)
Caproic acid (mg/L)	<DL (0.5 mg/L)	<DL (0.5 mg/L)	<DL (0.5 mg/L)	<DL (0.5 mg/L)

Acronyms: T-Alk: total alkalinity, VFA: volatile fatty acids, PT –P_2_O_5_: total phosphorous, TS: total solids, MS: mineral solids, VS: volatile solids, N-NH4+: ammoniacal nitrogen, TKN: total Kjeldahl Nitrogen, DL: Detection Limit, *Phaeodactylum Tricornutum* (*P. tricornutum*).

**Table 6:** Characteristics of the anaerobic inoculum used in the experiments.

Variables	Value	Variables	Value
pH	7.70 ± 0.01	TS (mg/L)	17,565 ± 115
Conductivity (mS/cm)	18.01 ± 0.01	MS (mg/L)	11,855 ± 110
T-Alk (mg CO3Ca/L)	9496 ± 65	VS (mg/L)	5710 ± 95
VFA (mg C/L)	195 ± 10	N-NH4+ (mg/L)	1055 ± 6
VFA (mg CH3COOH/L)	485 ± 25	N-TKN (mg/L)	1579 ± 10
PT –P2O5(mg/L)	16.60 ± 0.08	Acetic acid (mg/L)	54.48 ± 8.60
Cu (mg/L)	0.310 ± 0.002	Propionic acid (mg/L)	8.64 ± 2.35
Ni (mg/L)	0.450 ± 0.002	Isobutyric acid (mg/L)	9.67 ± 5.25
Zn (mg/L)	2.750 ± 0.030	Butyric acid (mg/L)	19.40 ± 5.40
Pb (mg/L)	0.310 ± 0.025	Isovaleric acid (mg/L)	n.d.
Cr (mg/L)	0.078 ± 0.002	Valeric acid (mg/L)	n.d.
Cd (mg/L)	0.090 ± 0.002	Caproic acid (mg/L)	n.d.

T-Alk: total alkalinity, VFA: volatile fatty acids, P_T_ –P_2_O_5_: total phosphorous, TS: total solids, MS: mineral solids, VS: volatile solids, N-NH_4_^+^: ammoniacal nitrogen, N-TKN: total Kjeldahl Nitrogen and n.d.: no detected.

## Data Availability

Not applicable.

## References

[1] Lopez P.J., Desclés J., Allen A.E., Bowler C. (2005). Prospects in diatom research. Curr. Opin. Biotechnol..

[2] Villanova V., Fortunato A.E., Singh D., Bo D.D., Conte M., Obata T., Jouhet J., Fernie A.R., Marechal E., Falciatore A. (2017). Investigating mixotrophic metabolism in the model diatom Phaeodactylum tricornutum. Philos. Trans. R. Soc. B-Biol. Sci..

[3] Field C.B., Behrenfeld M.J., Randerson J.T., Falkowski P. (1998). Primary production of the biosphere: Integrating terrestrial and oceanic components. Science.

[4] Armbrust E.V. (2009). The life of diatoms in the world’s oceans. Nature.

[5] Dolch L.-J., Maréchal E. (2015). Inventory of Fatty Acid Desaturases in the Pennate Diatom Phaeodactylum tricornutum. Mar. Drugs.

[6] Lebeau T., Robert J.-M. (2003). Diatom cultivation and biotechnologically relevant products. Part II: Current and putative products. Appl. Microbiol. Biotechnol..

[7] Kuppusamy P., Soundharrajan I., Srigopalram S., Yusoff M.M., Maniam G.P., Govindan N., Choi K.C. (2017). Potential pharmaceutical and biomedical applications of Diatoms microalgae-An overview. Indian J. Geo Mar. Sci..

[8] Sharma N., Simon D.P., Diaz-Garza A.M., Fantino E., Messaabi A., Meddeb-Mouelhi F., Germain H., Desgagné-Penix I. (2021). Diatoms Biotechnology: Various Industrial Applications for a Greener Tomorrow. Front. Mar. Sci..

[9] Atalah E., Cruz C.M.H., Izquierdo M.S., Rosenlund G., Caballero M.J., Valencia A., Robaina L. (2007). Two microalgae Crypthecodinium cohnii and Phaeodactylum tricornutum as alternative source of essential fatty acids in starter feeds for seabream (Sparus aurata). Aquaculture.

[10] Kim S.M., Jung Y.-J., Kwon O.-N., Cha K.H., Um B.-H., Chung D., Pan C.-H. (2012). A potential commercial source of fucoxanthin extracted from the microalga Phaeodactylum tricornutum. Appl. Biochem. Biotechnol..

[11] Eilers U., Bikoulis A., Breitenbach J., Büchel C., Sandmann G. (2016). Limitations in the biosynthesis of fucoxanthin as targets for genetic engineering in Phaeodactylum tricornutum. J. Appl. Phycol..

[12] Miyashita K., Nishikawa S., Beppu F., Tsukui T., Abe M., Hosokawa M. (2011). The allenic carotenoid fucoxanthin, a novel marine nutraceutical from brown seaweeds. J. Sci. Food Agric..

[13] Maeda H., Hosokawa M., Sashima T., Funayama K., Miyashita K. (2005). Fucoxanthin from edible seaweed, Undaria pinnatifida, shows antiobesity effect through UCP1 expression in white adipose tissues. Biochem. Biophys. Res. Commun..

[14] Sachindra N.M., Sato E., Maeda H., Hosokawa M., Niwano Y., Kohno M., Miyashita K. (2007). Radical scavenging and singlet oxygen quenching activity of marine carotenoid fucoxanthin and its metabolites. J. Agric. Food Chem..

[15] Guo B., Liu B., Yang B., Sun P., Lu X., Liu J., Chen F. (2016). Screening of diatom strains and characterization of Cyclotella cryptica as a potential fucoxanthin producer. Mar. Drugs.

[16] Poojary M.M., Barba F.J., Aliakbarian B., Donsì F., Pataro G., Dias D.A., Juliano P. (2016). Innovative alternative technologies to extract carotenoids from microalgae and seaweeds. Mar. Drugs.

[17] Chemat F., Vian M.A., Cravotto G. (2012). Green extraction of natural products: Concept and principles. Int. J. Mol. Sci..

[18] Armenta S., Garrigues S., Esteve-Turrillas F.A., de la Guardia M. (2019). Green extraction techniques in green analytical chemistry. Trac-Trends Anal. Chem..

[19] da Silva R.P.F.F., Rocha-Santos T.A.P., Duarte A.C. (2016). Supercritical fluid extraction of bioactive compounds. Trac-Trends Anal. Chem..

[20] Suganya T., Varman M., Masjuki H., Renganathan S. (2016). Macroalgae and microalgae as a potential source for commercial applications along with biofuels production: A biorefinery approach. Renew. Sust. Energ. Rev..

[21] Cherubini F. (2010). The biorefinery concept: Using biomass instead of oil for producing energy and chemicals. Energy Conv. Manag..

[22] Roadmap U. (2002). Roadmap for Biomass Technologies in the United States. Biomass Research and Development Technical Advisory Committee. https://biomassboard.gov/sites/default/files/pdfs/final_biomass_roadmap_2002kw.pdf.

[23] Naik S.N., Goud V.V., Rout P.K., Dalai A.K. (2010). Production of first and second generation biofuels: A comprehensive review. Renew. Sust. Energ. Rev..

[24] Gujer W., Zehnder A.J.B. (1983). Conversion Processes in Anaerobic Digestion. Water Sci. Technol..

[25] Passos F., Uggetti E., Carrère H., Ferrer I. (2014). Pretreatment of microalgae to improve biogas production: A review. Bioresour. Technol..

[26] Zhao B., Ma J., Zhao Q., Laurens L., Jarvis E., Chen S., Frear C. (2014). Efficient anaerobic digestion of whole microalgae and lipid-extracted microalgae residues for methane energy production. Bioresour. Technol..

[27] Lage S., Willfors A., Hörnberg A., Gentili F. (2021). Impact of organic solvents on lipid-extracted microalgae residues and wastewater sludge co-digestion. Bioresour. Technol. Reports.

[28] Sardessai Y.N., Bhosle S. (2004). Industrial potential of organic solvent tolerant bacteria. Biotechnology Progress.

[29] Cord-Ruwisch R. (2019). Thermodynamics of anaerobic digestion: Mechanism of suppression on biogas production during acidogenesis. INMATEH-Agric. Eng..

[30] Ruiz-Domínguez M.C., Marticorena P., Sepúlveda C., Salinas F., Cerezal P., Riquelme C. (2020). Effect of Drying Methods on Lutein Content and Recovery by Supercritical Extraction from the Microalga Muriellopsis sp. (MCH35) Cultivated in the Arid North of Chile. Mar. Drugs.

[31] Del Valle J.M., Uquiche E.L. (2002). Particle size effects on supercritical CO_2_ extraction of oil-containing seeds. J. Am. Oil Chem. Soc..

[32] Snyder J., Friedrich J., Christianson D. (1984). Effect of moisture and particle size on the extractability of oils from seeds with supercritical CO2. J. Am. Oil Chem. Soc..

[33] Reverchon E., Marrone C. (2001). Modeling and simulation of the supercritical CO2 extraction of vegetable oils. J. Supercrit. Fluids.

[34] Crampon C., Boutin O., Badens E. (2011). Supercritical carbon dioxide extraction of molecules of interest from microalgae and seaweeds. Ind. Eng. Chem. Res..

[35] Brunner G. (2013). Gas Extraction: An Introduction to Fundamentals of Supercritical Fluids and the Application to Separation Processes.

[36] Vieira de Melo S., Costa G.M., Viana A.C., Pessoa F.L. (2009). Computation of crossover pressure for synthesis of supercritical fluid separation systems. Comput. Chem. Eng..

[37] Vieira de Melo S., Costa G.M.N., Viana A., Pessoa F. (2009). Solid pure component property effects on modeling upper crossover pressure for supercritical fluid process synthesis: A case study for the separation of Annatto pigments using SC-CO_2_. J. Supercrit. Fluids.

[38] Rad H.B., Sabet J.K., Varaminian F. (2020). Study of solubility in supercritical fluids: Thermodynamic concepts and measurement methods-a review. Braz. J. Chem. Eng..

[39] Foster N.R., Gurdial G.S., Yun J.S., Liong K.K., Tilly K.D., Ting S.S., Singh H., Lee J.H. (1991). Significance of the crossover pressure in solid-supercritical fluid phase equilibria. Ind. Eng. Chem. Res..

[40] Fabrowska J., Ibañez E., Łęska B., Herrero M. (2016). Supercritical fluid extraction as a tool to valorize underexploited freshwater green algae. Algal Res..

[41] Ruiz-Domínguez M.C., Cerezal P., Salinas F., Medina E., Renato-Castro G. (2020). Application of Box-Behnken Design and Desirability Function for Green Prospection of Bioactive Compounds from Isochrysis galbana. Appl. Sci.-Basel.

[42] Roh M.K., Uddin M.S., Chun B.S. (2008). Extraction of Fucoxanthin and Polyphenol from Undaria pinnatifida Using Supercritical Carbon dioxide with Co-solvent. Biotechnol. Bioprocess Eng..

[43] Shi J., Mittal G., Kim E., Xue S.J. (2007). Solubility of Carotenoids in Supercritical CO2. Food Rev. Int..

[44] Kim S.M., Kang S.-W., Kwon O.-N., Chung D., Pan C.-H. (2012). Fucoxanthin as a major carotenoid in Isochrysis aff. galbana: Characterization of extraction for commercial application. J. Korean Soc. Appl. Biol. Chem..

[45] Zhang W., Wang F., Gao B., Huang L., Zhang C. (2018). An integrated biorefinery process: Stepwise extraction of fucoxanthin, eicosapentaenoic acid and chrysolaminarin from the same Phaeodactylum tricornutum biomass. Algal Res..

[46] Herrero M., Cifuentes A., Ibañez E. (2006). Sub-and supercritical fluid extraction of functional ingredients from different natural sources: Plants, food-by-products, algae and microalgae: A review. Food Chem..

[47] Gómez-Loredo A., Benavides J., Rito-Palomares M. (2014). Partition behavior of fucoxanthin in ethanol-potassium phosphate two-phase systems. J. Chem. Technol. Biotechnol..

[48] Conde E., Moure A., Domínguez H. (2015). Supercritical CO2 extraction of fatty acids, phenolics and fucoxanthin from freeze-dried Sargassum muticum. J. Appl. Phycol..

[49] Gilbert-López B., Barranco A., Herrero M., Cifuentes A., Ibáñez E. (2017). Development of new green processes for the recovery of bioactives from Phaeodactylum tricornutum. Food Res. Int..

[50] Mussgnug J.H., Klassen V., Schlüter A., Kruse O. (2010). Microalgae as substrates for fermentative biogas production in a combined biorefinery concept. J. Biotechnol..

[51] Sialve B., Bernet N., Bernard O. (2009). Anaerobic digestion of microalgae as a necessary step to make microalgal biodiesel sustainable. Biotechnol. Adv..

[52] Zabed H.M., Akter S., Yun J., Zhang G., Zhang Y., Qi X. (2020). Biogas from microalgae: Technologies, challenges and opportunities. Renew. Sust. Energ. Rev..

[53] Di Lena G., Casini I., Lucarini M., del Pulgar J.S., Aguzzi A., Caproni R., Gabrielli P., Lombardi-Boccia G. (2020). Chemical characterization and nutritional evaluation of microalgal biomass from large-scale production: A comparative study of five species. Eur. Food Res. Technol..

[54] Bernaerts T.M.M., Gheysen L., Kyomugasho C., Jamsazzadeh Kermani Z., Vandionant S., Foubert I., Hendrickx M.E., Van Loey A.M. (2018). Comparison of microalgal biomasses as functional food ingredients: Focus on the composition of cell wall related polysaccharides. Algal Res..

[55] Wu H., Li T., Wang G., Dai S., He H., Xiang W. (2016). A comparative analysis of fatty acid composition and fucoxanthin content in six Phaeodactylum tricornutum strains from diff erent origins. Chin. J. Oceanol. Limnol..

[56] Matos Â.P., Feller R., Moecke E.H.S., de Oliveira J.V., Junior A.F., Derner R.B., Sant’Anna E.S. (2016). Chemical Characterization of Six Microalgae with Potential Utility for Food Application. J. Am. Oil Chem. Soc..

[57] Niccolai A., Chini Zittelli G., Rodolfi L., Biondi N., Tredici M.R. (2019). Microalgae of interest as food source: Biochemical composition and digestibility. Algal Res..

[58] Rincón B., Jacob-Lopes E., Maroneze M.M., Queiroz M.I., Zepka L.Q. (2020). Chapter 12—Biogas from microalgae. Handbook of Microalgae-Based Processes and Products.

[59] Ramos-Suárez J.L., Carreras N. (2014). Use of microalgae residues for biogas production. Chem. Eng. J..

[60] Caporgno M.P., Olkiewicz M., Torras C., Salvadó J., Clavero E., Bengoa C. (2016). Effect of pre-treatments on the production of biofuels from Phaeodactylum tricornutum. J. Environ. Manag..

[61] Frigon J.-C., Abdou R.H., McGinn P.J., O’Leary S.J., Guiot S.R. (2014). Fate of palmitic, palmitoleic and eicosapentaenoic acids during anaerobic digestion of Phaeodactylum tricornutum at varying lipid concentration. Algal Res..

[62] Zamalloa C., Boon N., Verstraete W. (2012). Anaerobic digestibility of Scenedesmus obliquus and Phaeodactylum tricornutum under mesophilic and thermophilic conditions. Appl. Energy.

[63] Rittmann B.E., McCarty P.L. (2001). Biotecnología del Medio Ambiente, Principios y Aplicaciones.

[64] Zhang J., Wang S., Lang S., Xian P., Xie T. (2016). Kinetics of combined thermal pretreatment and anaerobic digestion of waste activated sludge from sugar and pulp industry. Chem. Eng. J..

[65] Chen Y., Cheng J.J., Creamer K.S. (2008). Inhibition of anaerobic digestion process: A review. Bioresour. Technol..

[66] Angelidaki I., Ahring B. (1993). Thermophilic anaerobic digestion of livestock waste: The effect of ammonia. Appl. Microbiol. Biotechnol..

[67] Hejnfelt A., Angelidaki I. (2009). Anaerobic digestion of slaughterhouse by-products. Biomass Bioenerg..

[68] Chong C.C., Cheng Y.W., Ishak S., Lam M.K., Lim J.W., Tan I.S., Show P.L., Lee K.T. (2022). Anaerobic digestate as a low-cost nutrient source for sustainable microalgae cultivation: A way forward through waste valorization approach. Sci. Total Environ..

[69] Guillard R.R. (1975). Culture of phytoplankton for feeding marine invertebrates. Culture of Marine Invertebrate Animals.

[70] American Society of Agricultural and Biological Engineers (2007). Method of Determining and Expressing Particle Size of Chopped Forage Materials by Screening.

[71] Salinas F., Vardanega R., Espinosa-Álvarez C., Jimenéz D., Muñoz W.B., Ruiz-Domínguez M.C., Meireles M.A.A., Cerezal-Mezquita P. (2020). Supercritical fluid extraction of chañar (Geoffroea decorticans) almond oil: Global yield, kinetics and oil characterization. J. Supercrit. Fluids.

[72] Rosa P.T., Meireles M.A.A. (2005). Rapid estimation of the manufacturing cost of extracts obtained by supercritical fluid extraction. J. Food Eng..

[73] del Valle J.M., Núñez G.A., Aravena R.I. (2014). Supercritical CO2 oilseed extraction in multi-vessel plants. 1. Minimization of operational cost. J. Supercrit. Fluids.

[74] Lowry O.H., Rosebrough N.J., Farr A.L., Randall R.J. (1951). Protein measurement with the Folin phenol reagent. J. Biol. Chem..

[75] Dubois M., Gilles K.A., Hamilton J.K., Rebers P.t., Smith F. (1956). Colorimetric method for determination of sugars and related substances. Anal. Chem..

[76] Geresh S., Adin I., Yarmolinsky E., Karpasas M. (2002). Characterization of the extracellular polysaccharide of Porphyridium sp.: Molecular weight determination and rheological properties. Carbohydr. Polym..

[77] Axelsson M., Gentili F. (2014). A Single-Step Method for Rapid Extraction of Total Lipids from Green Microalgae. PLoS ONE.

[78] Clesceri L.S., Greenberg A.E., Eaton A.D., Rice E.W., Franson M.A.H., APHA, AWWA, WPCF (2005). Standard Methods for the Examination of Water and Wastewater.

[79] The US Department of Agriculture and the US Composting Council (2002). Test Methods for the Examination of Composting and Compost (TMECC).

